# Fumarate-associated immunometabolic network in Periodontitis revealed by integrated transcriptomic and single-cell RNA sequencing analysis

**DOI:** 10.1371/journal.pone.0353794

**Published:** 2026-08-05

**Authors:** Rongpeng Liu, Jinfeng Lv, Yao Zeng, Jieping Wang, Xiaoyan Wang, Yang Liu, Shuo Li, Han Qin, Sumbal Zaheen, Zuohua Liu, Jinlin Song, Chan Zhou

**Affiliations:** 1 Chongqing Academy of Animal Sciences, Chongqing, China; 2 College of Stomatology, Chongqing Medical University, Chongqing, China; 3 Institute for Brain Sciences Research, School of Life Sciences, Henan University, Kaifeng, China; 4 Chongqing Key Laboratory of Oral Diseases, Chongqing, China; 5 Chongqing Municipal Key Laboratory of Oral Biomedical Engineering of Higher Education, Chongqing, China; 6 State Key Laboratory of Resource Insects, Southwest University, Chongqing, China; Versiti Blood Research Institute, UNITED STATES OF AMERICA

## Abstract

Periodontitis is a long-term inflammatory disease of the gums that damages the tissues and bone supporting the teeth. Recent studies suggest that changes in cellular metabolism may influence immune responses during chronic inflammation; however, the contribution of fumarate-associated transcriptional programs to periodontitis remains unclear. In this study, we integrated bulk transcriptomic data from human periodontal tissues with single-cell RNA sequencing data to identify fumarate-associated genes and cell clusters associated with periodontitis. Our analyses revealed a fumarate-associated immune regulatory signature that was enriched in periodontitis. Three genes, *C3*, *CXCR4*, and *MEF2C*, were consistently increased in diseased tissues and showed similar expression trends in a ligature-induced mouse model of periodontitis. These genes showed transcript-level diagnostic potential and were predominantly expressed in immune cells, including myeloid cells and neutrophils, as well as stromal cells. In addition, computational drug screening and molecular docking highlighted carbenoxolone and a CXCR4 antagonist (USL311) as potential candidates for future study. Overall, this study provides an integrative view of fumarate-associated immunometabolic alterations in periodontitis and identifies candidate transcriptomic markers and therapeutic hypotheses that warrant further experimental validation.

## Introduction

Periodontitis (PD) is a long-standing inflammatory disease that affects the gingiva and supporting periodontal structures, leading to progressive, irreversible destruction of the alveolar bone and periodontal ligament. It is a major global public health problem affecting billions worldwide and a leading cause of tooth loss and compromised oral health [1]. These conditions are closely associated with several disorders, including diabetes [2], cardiovascular diseases [3], and Alzheimer’s disease [4]. Its pathogenicity extends beyond microbial biofilms to encompass a dysregulated, ongoing host immune response [5–7]. Sustained local inflammation disrupts metabolism at the site. Immunometabolism research now reveals that metabolic reprogramming actively drives the fate and function of immune cells [8–11], a key mechanism underlying chronic inflammation, including PD.

The TCA cycle occupies a central position in cellular metabolism [12–14]. Beyond classical metabolic functions, intermediates such as fumarate perform potent signaling and immunomodulatory functions [15–18]. The accumulation of fumarate can stabilize hypoxia-inducible factor 1α (HIF-1α) and promote protein succination, thereby directly affecting immune cell transcription and inflammatory responses [19–23]. Although fumarate has been implicated in the mechanisms underlying other inflammatory diseases [24–27], its precise role in the immunopathogenesis of PD remains to be established.

To address this gap, we used an integrative multi-omics strategy to evaluate fumarate-associated alterations in immune networks in periodontitis. This exploratory framework combined bulk RNA sequencing and high-resolution single-cell transcriptomic data with bioinformatics and machine-learning methods [28–30]. Our analysis identified an inferred fumarate-associated gene set containing C3, CXCR4, and MEF2C. These genes were elevated in PD, showed transcript-level diagnostic potential across public datasets, and were expressed in myeloid/neutrophil and stromal niches. In addition, computational drug screening and molecular docking identified candidate compounds for future follow-up studies. Overall, our findings define a fumarate-associated immunometabolic signature in periodontitis and nominate C3, CXCR4, and MEF2C as candidate transcriptomic markers for further mechanistic and translational validation.

## Materials and methods

### Data acquisition

The scRNA-seq dataset GSE164241 and the bulk RNA-seq datasets GSE16134 (training set) and GSE10334 (validation set) were retrieved from the GEO database (https://www.ncbi.nlm.nih.gov/geo). The scRNA-seq dataset GSE164241 includes periodontal tissue samples from 13 healthy individuals and 8 untreated PD patients [31,32]. GSE16134 comprises 310 samples (69 healthy individuals vs. 241 PD patients) [33], and GSE10334 consists of 247 samples (64 healthy individuals vs. 183 PD patients) [34]. To capture a broad exploratory set of fumarate-associated candidates, 1134 fumarate-related genes (FRGs) were obtained from the GeneCards database using the keyword “fumaric” (https://www.genecards.org/) [35]. No GeneCards relevance-score cutoff was applied; all genes returned by this keyword search were retained for the initial screen. We therefore treated the FRG set as an exploratory fumarate-associated candidate pool rather than a curated list of genes directly participating in the TCA cycle or fumarate metabolism, and we considered this potential source of biological heterogeneity when interpreting downstream results. All datasets were processed to remove batch effects using the sva R package (v4.5.1) before downstream analysis.

### Transcriptomic analysis

#### Identification of differentially expressed genes (DEGs).

DEGs between PD patients and controls in GSE16134 were identified using the limma R package (v1.38.0) with thresholds of adjusted p-value < 0.05 and |log2fold change (FC)| > 0.5. The fold-change cutoff was selected to balance biological interpretability with sensitivity in heterogeneous public tissue datasets and to retain moderate but potentially relevant transcriptomic changes for downstream network analysis. Volcano plots and heatmaps were generated with ggplot2 (v3.3.6) and ComplexHeatmap (v2.14.0). GO (biological processes, cellular components, molecular functions) and KEGG pathway enrichment analyses were performed using clusterProfiler (v4.7.1.3) and org.Hs.e.g.,db (3.14.0), with p-value < 0.05 as the significance threshold [36,37].

#### Weighted gene co-expression network analysis (WGCNA).

Genes with median expression > 0.5 in GSE16134 were used for WGCNA (WGCNA R package) [38]. The optimal soft threshold was determined using scale-free topology analysis (R² = 0.85). Gene co-expression modules were constructed via hierarchical clustering of the topological overlap matrix (TOM) using topological overlap as the similarity metric, with a minimum module size of 100 and a merge cut height of 0.25. Module-trait correlation analysis was performed to identify modules significantly associated with PD (p < 0.05), and core module genes were defined as those with module membership (MM) > 0.5 and gene significance (GS) > 0.5.

#### Candidate gene screening.

Candidate genes were identified by intersecting three gene sets: PD-specific DEGs, fumarate-related genes (FRGs) [35], and WGCNA module genes passing the predefined MM/GS thresholds. The intersection was visualized using the Venn Diagram R package (version 1.7.3), and functional enrichment analysis of the candidate genes was performed using the clusterProfiler R package. A PPI network was constructed using the STRING database (confidence score > 0.4) and visualized with Cytoscape (v3.9.1).

#### Machine learning-based feature selection.

The least absolute shrinkage and selection operator (LASSO) regression model and support vector machine recursive feature elimination (SVM-RFE) [39] were used to select the most relevant key genes. Transcript-level discriminatory performance was evaluated using ROC curves and AUC values via the pROC R package (v1.18.0) on both the training and validation sets.

### Expression validation

#### Clinical dataset validation.

To validate the robustness and clinical relevance of the identified key genes, independent transcriptomic datasets were employed. Gene expression levels from GSE16134 were used as the training cohort, and those from GSE10334 as the external validation cohort. Both datasets comprised tissue samples obtained from patients with PD, and the control groups were compared using the Wilcoxon rank-sum test (p < 0.05 considered significant).

#### Animal model and qRT-PCR validation.

To provide experimental support for the transcriptomic findings, a ligature-induced mouse model of PD was established. Briefly, 6-week-old male C57BL/6 mice (18–20 g) were randomly divided into PD and control groups (n = 3 per group). The animals were acclimated for 7 days before the experiment and housed under specific-pathogen-free conditions with a 12 h light/dark cycle and free access to food and water. This pilot animal experiment was designed for exploratory qRT-PCR validation, and no a priori sample-size calculation was performed. Each mouse was treated as one biological replicate. PD was induced by placing a 5–0 silk suture around the maxillary 2nd molar under intraperitoneal 1.25% tribromoethanol (approximately 250 mg/kg body weight) anesthesia. Adequate anesthesia was confirmed by the absence of pedal withdrawal and corneal reflexes before manipulation. The oral cavity was opened gently, and the ligature was passed through the interproximal spaces around the maxillary second molar and tied on the buccal side. Excess suture was trimmed to minimize irritation and reduce the risk of ligature displacement. The procedure was performed carefully to avoid unnecessary gingival trauma. Control mice did not receive ligature placement. During the 10-day experimental period, animals were monitored daily for general condition, body weight, food intake, grooming behavior, signs of distress, and ligature retention. Ligature stability was assessed by visual inspection. Complete ligature loss before the endpoint, severe local trauma, abnormal bleeding, marked weight loss, or poor general condition were predefined exclusion criteria. If a ligature became clearly displaced but remained around the target molar, its position was recorded, and the animal was retained only when the ligature was still functionally present at the target site. No sample was treated as an independent biological replicate unless it came from a different mouse. The 10-day endpoint was selected to capture an active inflammatory phase suitable for transcript-level validation. Previous ligature-induced periodontitis models in C57BL/6 mice have shown that gingival inflammation and alveolar bone loss develop within days after ligature placement, with progressive periodontal tissue changes occurring during the first 1–2 weeks [40]. Because the purpose of this experiment was to validate expression trends of selected genes rather than to perform late-stage histomorphometric or therapeutic evaluation, a 10-day induction period was considered appropriate [40]. At the endpoint, mice were deeply anesthetized with 1.25% tribromoethanol and euthanized by cervical dislocation under deep anesthesia. Maxillary gingival/periodontal tissues surrounding the ligated second molar were collected from periodontitis mice. Anatomically matched maxillary periodontal tissues were collected from untreated control mice. To avoid pseudo-replication and intra-animal correlation, only one predefined periodontal tissue sample from each mouse was used for qRT-PCR analysis; contralateral sites, if present, were not analyzed as independent samples. Tissues were rapidly dissected using sterile instruments, immediately preserved for RNA extraction, and processed using TRIzol reagent (TaKaRa, Japan) according to the manufacturer’s protocol. RNA concentration and purity were assessed spectrophotometrically, and an equal amount of RNA was reverse transcribed into cDNA using the PrimeScript RT Kit (TaKaRa). qRT-PCR was performed using UltraSYBR Mixture (CWBIO, China) on a QuantStudio 6 Flex Real-Time PCR System. GAPDH was used as the internal reference gene. Relative gene expression levels were calculated using the 2^⁻ΔΔCt^ method. Each qRT-PCR reaction was performed in technical triplicate. The mean Ct value of the technical triplicates was used to generate one expression value for each mouse, and statistical comparisons were performed using mouse-level biological replicates. Primer sequences are listed in S8 Table. All surgeries were performed after anesthesia, and every effort was made to minimize pain in the mice. Animal reporting was prepared with reference to the ARRIVE 2.0 recommendations where applicable. All animal experiments were approved by the Chongqing Academy of Animal Science Animal Ethics Committee (Ethics number: XKY-20270201). The study was conducted in accordance with the local legislation and institutional requirements.

### Functional and regulatory mechanism analysis

#### Gene Set Enrichment Analysis (GSEA).

GSEA was performed to identify biological pathways associated with key gene expression patterns. The analysis was performed using the clusterProfiler R package (v4.7.1.3). KEGG pathway gene sets were used as the reference database to identify pathways associated with key gene-expression patterns. Statistical significance was determined using permutation testing, and pathways with p < 0.05 and |normalized enrichment score (NES)| > 1 were considered significantly enriched.

#### ceRNA network construction.

To investigate the potential post-transcriptional regulatory mechanisms involving key genes, a ceRNA network was constructed. Upstream miRNAs targeting key genes were predicted using the miRanda (v3.3a) and miRDB (v6.0) databases via the multiMiR R package (v1.22.0). Subsequently, upstream lncRNAs that potentially interact with the identified were retrieved from StarBase (v3.0), which integrates experimentally validated and computationally predicted RNA-RNA interaction data. The ceRNA network was visualized using the ggraph R package (v2.1.0) to optimize the layout. Final network visualization, refinement, and topological analysis were performed using Cytoscape (v3.9.1).

#### Immune infiltration analysis (CIBERSORT).

GSE16134 was analyzed using CIBERSORT (v1.03) as previously described by [41] with the LM22 reference matrix. Samples with CIBERSORT p < 0.05 were retained. The relative abundance of 22 immune cell types was compared between the PD and control groups using the Wilcoxon rank-sum test. Correlations between key gene expression and immune cell abundance were analyzed via Spearman’s correlation (p < 0.05 considered significant).

#### Chromosome localization analysis (OmicCircos).

The Chromosomal distribution of the key genes *C3*, *CXCR4*, and *MEF2C* was analyzed and visualized using the OmicCircos Package (v1.48.0) in R [42]. Human genome coordinates for these genes were obtained from the Ensembl database using the human reference genome assembly GRCh38.

### Single-cell RNA-Seq analysis

#### Data processing and cell annotation.

scRNA-seq data (GSE164241) were processed with Seurat (v5.0.1). Cells with 200–3000 detected genes and mitochondrial gene content < 15% were retained. Data were normalized with SCTransform, and 2000 highly variable genes were selected for PCA. Cells were clustered using the top 30 PCs at a resolution of 0.4, and the resulting clusters were visualized with a UMAP plot. Cell types were annotated using canonical marker genes.

#### Sub-clustering and pseudotime trajectory analysis.

To further investigate intra-population heterogeneity, key cell clusters identified in the initial clustering analysis were extracted and re-clustered. Pseudotime trajectory analysis was performed using Monocle 2 (v2.30.1) as previously described by [43] to reconstruct potential cell differentiation trajectories and infer dynamic cellular state transitions, with key gene expression visualized along the trajectory.

#### Cell-cell communication analysis.

Cell-cell communication networks were constructed using CellChat (v1.6.1) and the CellChatDB.human database, as described previously [44]. Communication probability and interaction strength between cell types were calculated, and ligand-receptor pairs involving key genes (e.g., CXCR4-CXCL12, C3-ITGAX/ITGB2) were prioritized. Differences in cell communication between PD and control groups were compared using permutation tests (p < 0.05).

#### Drug sensitivity and molecular docking analysis.

Potential therapeutic drugs targeting key genes were screened from the DGIdb database (https://www.dgidb.org). 3D structures of drugs and target proteins were retrieved from PubChem and PDB, respectively. Protein preparation (water removal and hydrogen addition) was performed using PyMOL (v3.0.3), and docking simulations, as previously described [45], were performed with AutoDock (v1.5.7). Binding energies < −5.0 kcal/mol were considered significant, and docking results were visualized in PyMOL.

### Statistical analysis

Statistical analyses were performed using R (v4.3.1) and GraphPad Prism (v9.0). For public transcriptomic validation analyses and comparisons of immune-cell proportions, nonparametric Wilcoxon rank-sum tests were used. For qRT-PCR data, normality was assessed before two-group comparisons, and Student’s t-test was applied when parametric assumptions were considered acceptable for this pilot experiment. Correlations between key gene expression and immune-cell abundance were assessed using Spearman’s rank correlation. Differential expression analysis used multiple-testing correction as implemented in the limma workflow, and an adjusted p < 0.05 was considered significant unless otherwise specified. Quantitative data are presented as mean ± SEM unless otherwise stated. A p-value < 0.05 was considered statistically significant (*, p < 0.05; **, p < 0.01; ***, p < 0.001; ****, p < 0.0001).

## Results

### Transcriptomic profiling reveals molecular signatures of PD

**Fig 1:**
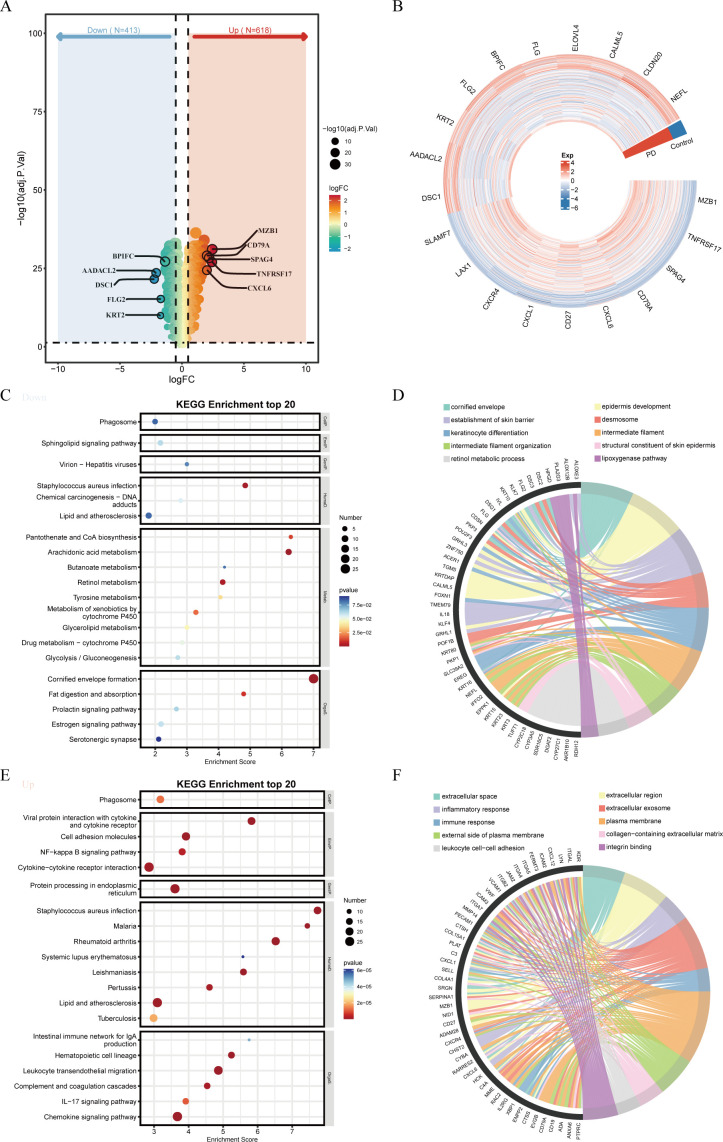
Differential expression and functional enrichment analysis in PD. (A) Volcano plot of DEGs between the control and PD groups. Red and blue dots denote up-regulated and down-regulated genes, respectively; the top five significantly altered genes are labeled. (B) Heatmap of the top 10 DEGs between the control and PD groups. (C) Top 20 enriched KEGG pathways for down-regulated DEGs. (D) GO enrichment chord diagram for down-regulated DEGs. (E) Top 20 enriched KEGG pathways for up-regulated DEGs. (F) GO enrichment chord diagram for up-regulated DEGs.

The training dataset GSE16134 (241 PD versus 69 controls) was analyzed using bulk RNA sequencing to characterize the molecular landscape of PD. Differential expression analysis identified 1,031 differentially expressed genes (DEGs) (Figs 1A and 1B; S1 Table). Of these, 618 were upregulated and 413 were downregulated.

Functional enrichment analysis indicated that down-regulated DEGs were primarily associated with epidermal barrier integrity (e.g., cornified envelope, keratinocyte differentiation) and metabolic pathways (e.g., retinol metabolism, lipoxygenase activity) (Figs 1C and 1D; S2 Table). Conversely, up-regulated DEGs were enriched in immune activation processes (e.g., inflammatory response, leukocyte cell-cell adhesion) and cell adhesion molecules (CAMs) (Figs 1E and 1F; S2 Table). Collectively, this transcriptional profile implicates concurrent impairment of barrier function, metabolic disturbance, and pronounced immune dysregulation in PD.

### WGCNA identifies co-expression modules associated with PD

**Fig 2:**
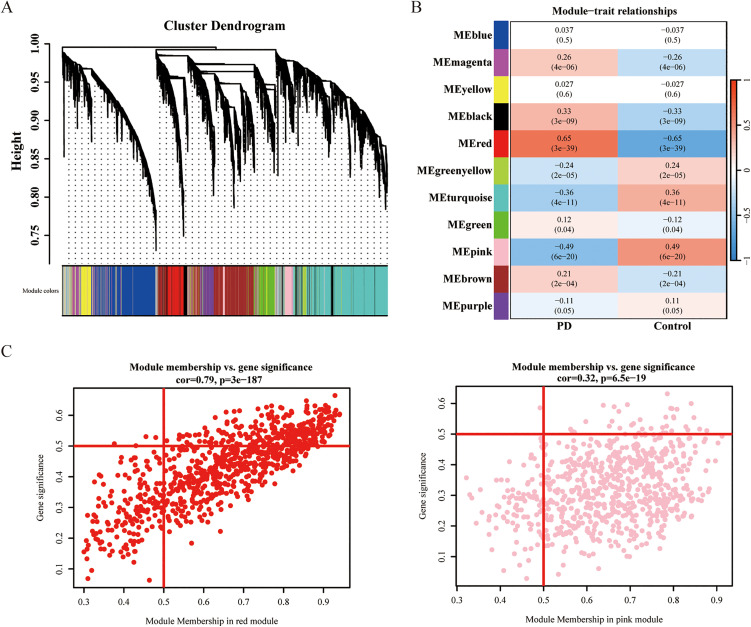
WGCNA identifies PD-associated gene modules. (A) Hierarchical clustering dendrogram of genes. (B) Heatmap of module–trait correlations. Color intensity represents Pearson correlation coefficients; values indicate correlation (top) and p-value (bottom). MEred and MEpink show significant correlations with PD. (C) Scatter plots of MM versus GS for the red (left) and pink (right) modules. Strong correlations indicate that highly connected module genes are also strongly associated with PD.

To identify gene modules correlated with PD, we performed weighted gene co-expression network analysis (WGCNA) on 16,183 filtered genes from GSE16134. Using a soft-threshold power of 18 (scale-free R^2^ = 0.85), we identified 11 distinct co-expression modules (Figs 2A and 2B; S1A and S1B Figs). Module-trait correlation analysis revealed that the red and pink modules exhibited the strongest associations with PD (Fig 2B). High correlations between gene significance (GS) for PD and module membership (MM) were observed in both modules (Fig 2C), supporting the biological relevance of these modules for downstream exploratory analysis. We thereby defined 309 genes from these two modules as genes associated with the inferred PD-related co-expression modules (S3 Table).

### Identification and functional annotation of candidate genes

**Fig 3:**
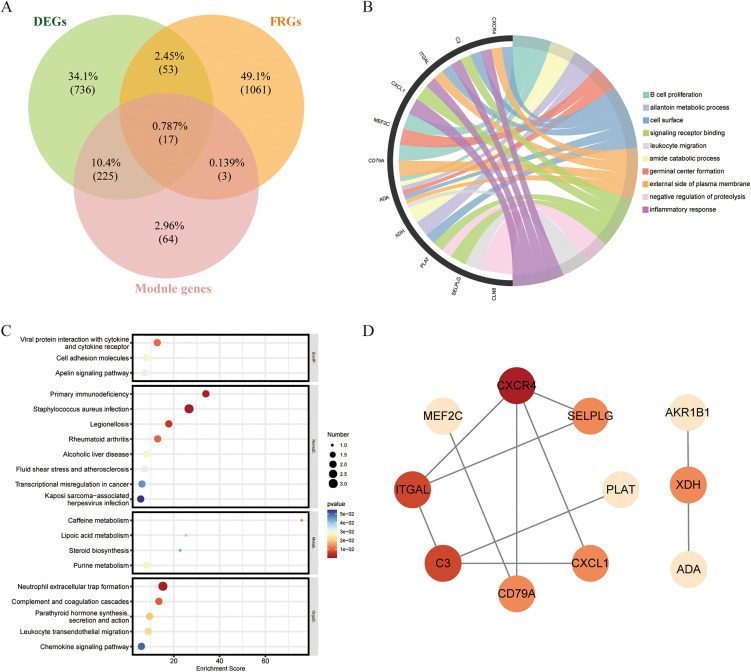
Screening and functional analysis of candidate genes. (A) Venn diagram of DEGs, FRGs, and module genes. (B) GO enrichment chord diagram of the 17 candidate genes. (C) KEGG pathway enrichment of the 17 candidate genes. (D) PPI network of the candidate genes. Highly connected genes are emphasized to reflect their relative connectivity within the inferred PPI network.

To identify fumarate-associated transcriptomic features in PD, we focused on the intersection of three key sets: PD-specific DEGs (n = 1,031), fumarate-related genes (FRGs, n = 1,134; see Methods), and genes from the PD-associated WGCNA modules (module genes, n = 309) (Figs 1A, 2B and 2C; S1, S3 and S4 Tables). This integrative filter yielded 17 candidate genes (Fig 3A; S5 Table), including *CXCR4*, *C3*, *MEF2C*, *CD79A*, and *PLAT*.

Functional enrichment analysis implicated these genes in B cell proliferation, inflammatory response, and the CAMs pathway (Figs 3B and 3C; S6 Table). Protein-Protein Interaction (PPI) network analysis highlighted *CXCR4*, *C3*, *CD79A*, *ITGAL,* and *XDH* as highly connected genes within the inferred PPI network (Fig 3D), suggesting their potential relevance within the PD-associated immunometabolic gene set.

### Machine learning identifies transcriptomic markers associated with PD

**Fig 4:**
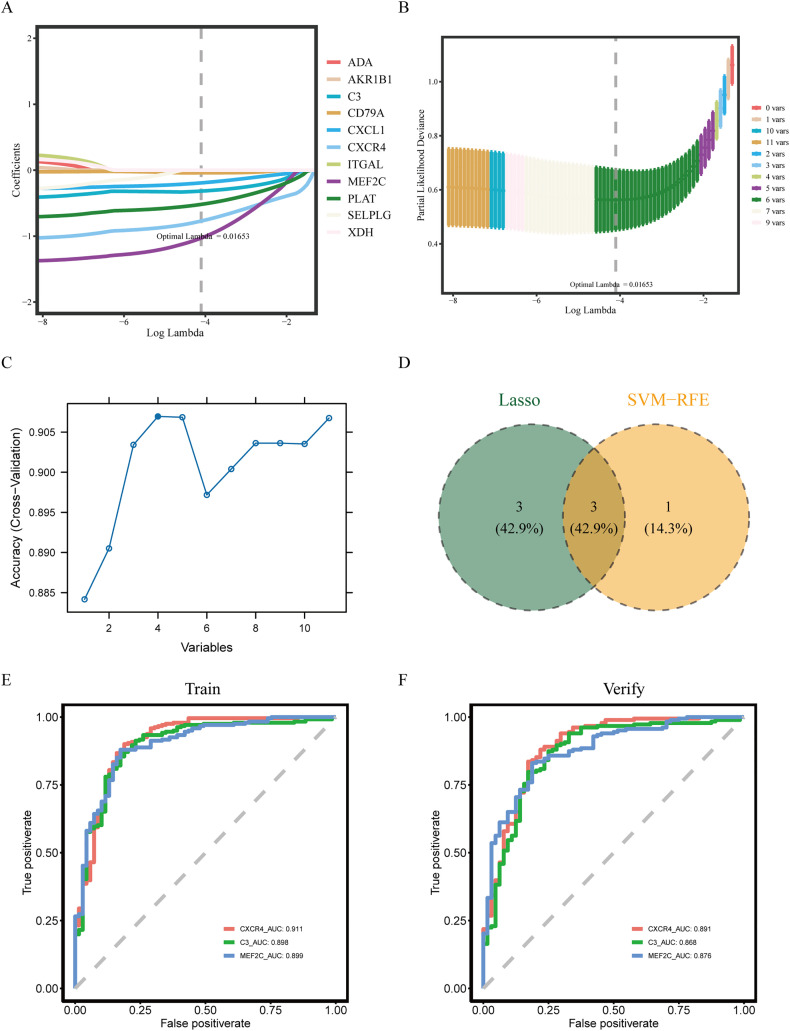
Machine learning-based transcriptomic marker screening. (A) LASSO coefficient trajectories. (B) Cross-validation curve for LASSO. (C) SVM-RFE analysis accuracy curve. (D) Venn diagram of genes selected by LASSO and SVM-RFE. (E) ROC curves of key genes in the training set. (F) ROC curve of key genes in the validation set.

To identify transcriptomic features associated with PD, we subjected the 17 candidate genes to a machine-learning analysis that combined LASSO regression and SVM-RFE. This dual approach converged on three consensus genes: C3, CXCR4, and MEF2C (Figs 4A-4D; S7 Table). ROC analysis showed good transcript-level discrimination for these genes in both the training set (GSE16134; AUC: C3 = 0.898, CXCR4 = 0.911, MEF2C = 0.899) and an independent validation set (GSE10334; AUC: C3 = 0.868, CXCR4 = 0.891, MEF2C = 0.876) (Figs 4E and 4F), supporting their potential as candidate transcriptomic markers associated with PD.

### Validation of key gene expression

**Fig 5:**
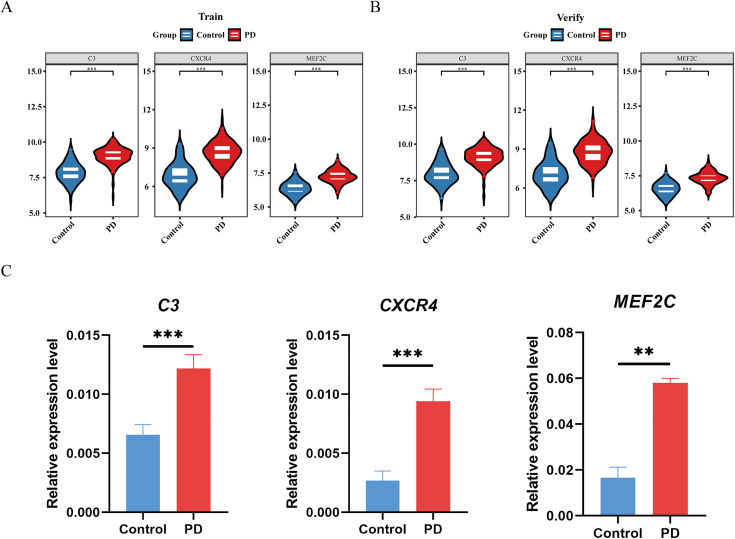
Expression validation of key genes. (A) Box plots of gene expression in the training set. (B) Box plots of gene expression in the validation set. (C) qRT-PCR results in a small exploratory mouse PD model. Data are presented as mean ± SD. *, *p* < 0.05; **, *p* < 0.01; ***, *p* < 0.001; ****, *p* < 0.0001.

The expression levels of *C3*, *CXCR4*, and *MEF2C* were systematically evaluated in both the training and validation cohorts. As shown in Fig 5A, the violin plot analysis of the training dataset revealed that all three genes were significantly higher in PD samples than in controls, with clear separation of expression distributions and consistent statistical significance. These findings were independently validated in an external dataset, where similarly elevated expression levels of *C3*, *CXCR4*, and *MEF2C* were observed in the PD sample (Fig 5B). To further corroborate these bioinformatic observations, qRT-PCR was performed in a small exploratory mouse model of PD. Consistent with the transcriptomic analyses, mouse-level qRT-PCR results showed increased expression of *C3*, *CXCR4*, and *MEF2C* (Fig 5C; S8 Table).

### Immune landscape and genomic localization

**Fig 6:**
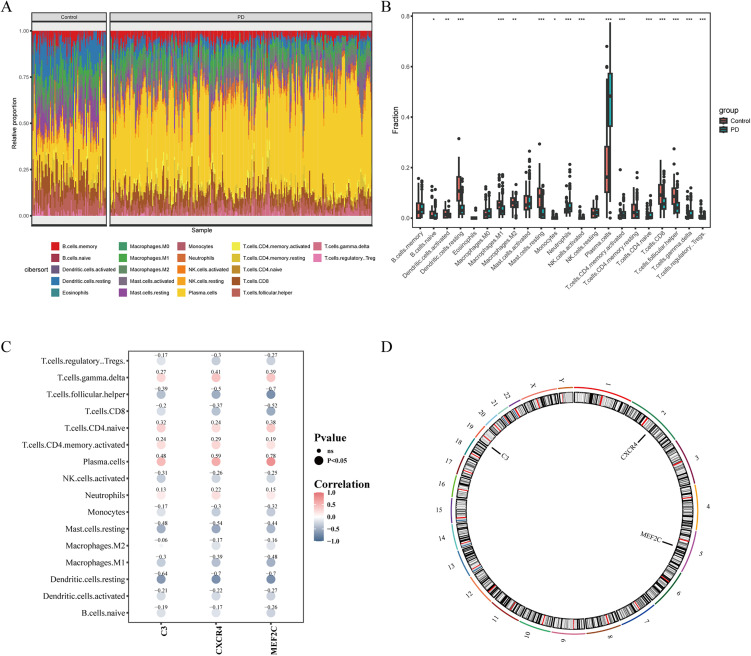
Immune infiltration profiles and chromosomal localization of key genes. (A) Heatmap of immune cell infiltration. (B) Comparative abundance of immune cell types. (C) Correlation heatmap between key genes and immune cell types. (D) Circular plot showing chromosomal locations of key genes.

CIBERSORT-based immune infiltration analysis revealed significant differences in immune cell composition between PD and control tissues. Compared to healthy controls, PD samples exhibited a significant increase in neutrophils, plasma cells, activated memory CD4^+^ T cells, naive CD4^+^ T cells, and γδ T cells. In contrast, the proportions of naive B cells, CD8^+^ T cells, resting and activated dendritic cells (DCs), M1 and M2 macrophages, resting mast cells, follicular helper T (Tfh) cells, and regulatory T (Treg) cells were significantly reduced in PD tissues (Figs 6A and 6B; S9 Table).

Spearman’s correlation analysis further demonstrated that *C3* expression positively correlated with plasma cells and naive CD4^+^ T cells, whereas *CXCR4* and *MEF2C* expression showed a positive correlation with plasma cells and γδ T cells (Fig 6C; S10 Table). Finally, genomic localization analysis confirmed that these core genes are located on chromosomes 19 (C3), 2 (*CXCR4*), and 5 (*MEF2C*), respectively (Fig 6D).

### Functional pathway enrichment and ceRNA network analysis

**Fig 7:**
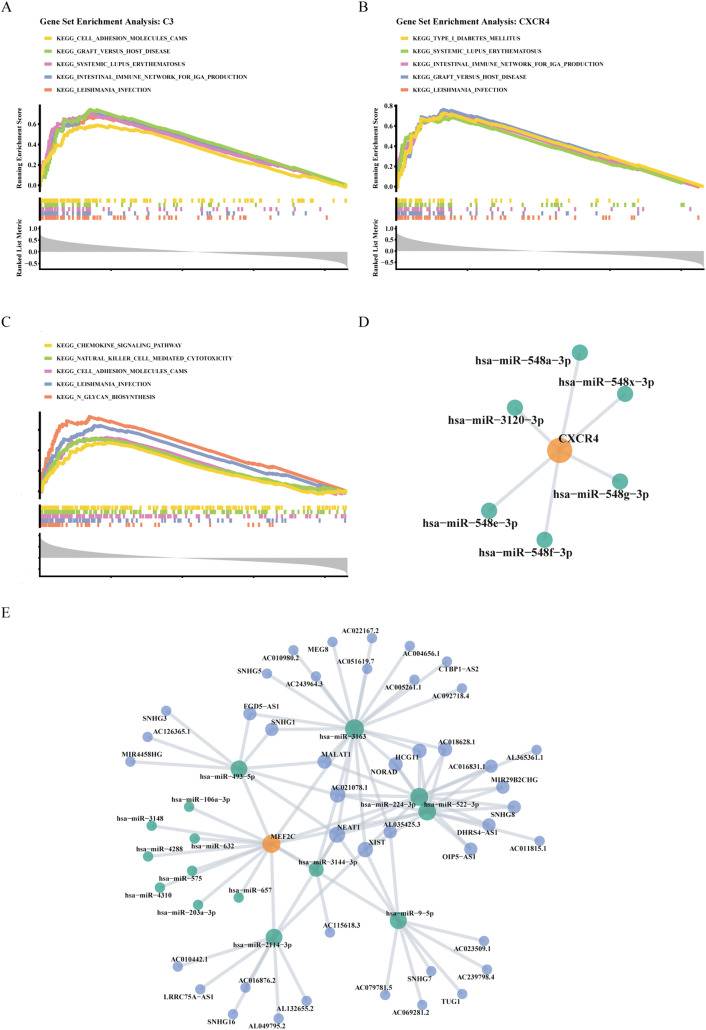
GSEA and ceRNA regulatory networks of key genes. (A) GSEA enrichment plots for *C3*. (B) GSEA enrichment plots for *CXCR4*. (C) GSEA enrichment plots for *MEF2C*. (D) ceRNA regulatory network for *CXCR4*. (E) ceRNA regulatory network for *MEF2C*.

Gene Set Enrichment Analysis (GSEA) delineated pathways associated with the three identified transcriptomic features. Both *C3* and *CXCR4* were predominantly enriched in immune and inflammatory pathways, including CAMs, autoimmune diseases, and infection responses. In contrast, *MEF2C* was specifically associated with chemokine signaling, cytotoxicity, and glycosylation pathways (Figs 7A-7C; S11 Table).

To further investigate the post-transcriptional regulatory mechanisms of these genes, a competing endogenous RNA (ceRNA) network was constructed. The analysis identified multiple upstream regulatory miRNAs targeting *CXCR4* (6 miRNAs) and *MEF2C* (15 miRNAs), whereas no candidate miRNAs were predicted for *C3* (Figs 7D and 7E; S12 Table). These findings suggest that CXCR4 and MEF2C may be subject to complex post-transcriptional regulation, whereas C3 expression may be primarily governed by an alternative regulatory mechanism in PD.

### Single-cell atlas reveals microenvironment remodeling in PD

**Fig 8:**
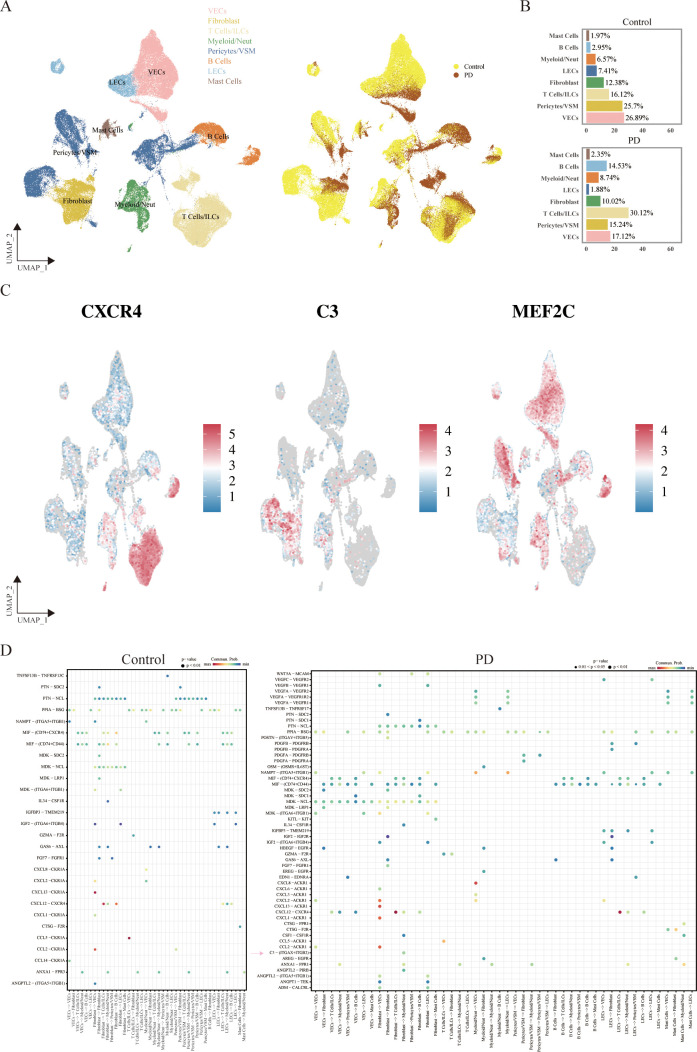
Single-cell analysis of PD tissues. (A) UMAP of cell types (left) and groups (right). (B) Relative abundance of cell types in control and PD. (C) UMAP expression maps for *CXCR4*, *C3*, and *MEF2C*. (D) Cell-cell communication networks in control (left) and PD (right).

To deconvolve the periodontal microenvironment, we performed single-cell RNA sequencing on patient tissues and, after strict quality control, analyzed a total of 130,447 high-quality cells (S2A-S2F Figs). We first defined the cellular landscape, identifying eight major cell types: myeloid/neutrophils (Myeloid/Neut), T cells/ILCs, pericytes/vascular smooth muscle cells (Pericytes/VSM), B cells, lymphatic endothelial cells (LECs), fibroblasts, vascular endothelial cells (VECs), and mast cells (Fig 8A; S3A-S3C Figs). In PD, this landscape shifted toward an immune-inflamed state, characterized by an increased number of immune cells (T cells/ILCs, B cells) and a decreased number of stromal/vascular components (VECs, Pericytes/VSM, LECs) (Fig 8B; S3D Fig; S13 Table).

Our three key transcriptomic markers exhibited cell-type-specific expression patterns. *C3* was predominantly expressed by stromal and myeloid cells (Pericytes/VSM, Myeloid/Neut, and fibroblasts); *CXCR4* was expressed by lymphoid and myeloid lineages (T cells/ILCs, Myeloid/Neut, and B cells); and *MEF2C* was broadly expressed across vascular, stromal, and immune subsets (VECs, LECs, Myeloid/Neut, Pericytes/VSM, and B cells). Their expression was higher in PD in these respective niches (Fig 8C; S3E-S3G Figs).

Cell-cell communication analysis using CellChat predicted enhanced immune-stromal crosstalk, especially between Myeloid/Neut and LECs in PD via several ligand-receptor pairs, including NAMPT-(ITGA5 + ITGB1) and PPIA-BSG (Fig 8D; S4A-S4D Figs; S14 Table). Importantly, CellChat predicted a PD-enriched potential communication pattern between fibroblasts and myeloid/neutrophil cells via C3-(ITGAX+ITGB2) (Fig 8D), along with increased signaling between myeloid cells and lymphatic endothelia.

Given the prominent involvement of the Myeloid/Neut compartment, subclustering and pseudo-time analysis of Myeloid/Neut cells identified 14 subclusters, with cluster (C) 9 representing an early inferred trajectory state (S5A-S5C, S6A and S6B Figs). Pseudo-time ordering revealed eleven differentiation states, with state 1 showing reduced representation in PD (S6C and S6D Figs). The dynamic expression patterns along the trajectory suggested that *C3* and *MEF2C* were more highly expressed in earlier inferred states, whereas *CXCR4* was associated with later inferred states (S6E and S6F Figs). Thus, these factors appear to be associated with different inferred myeloid/neutrophil trajectory states in PD.

### Drug screening and molecular docking

**Fig 9:**
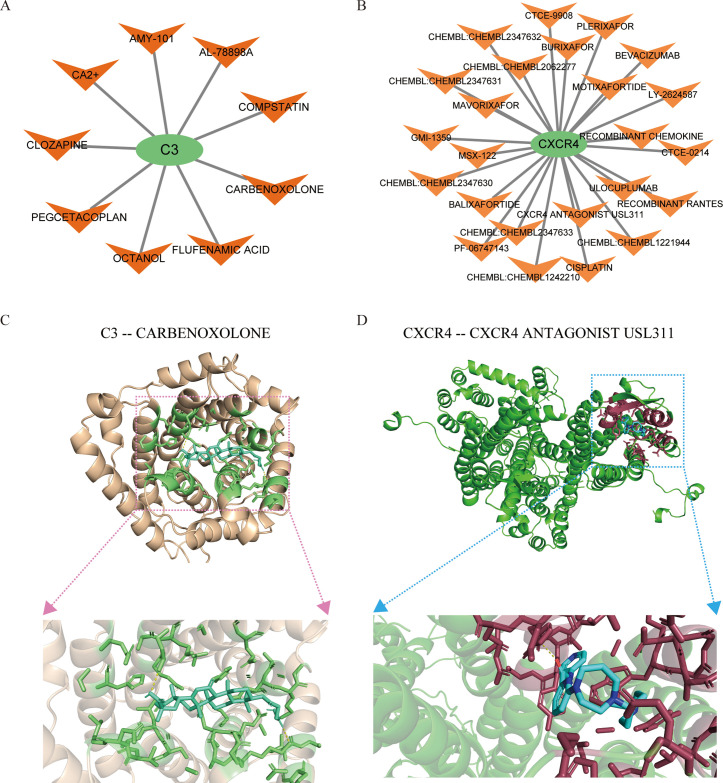
Therapeutic drug screening and docking validation. (A) Radial plots of candidate drugs for *C3*. (B) Radial plots of candidate drugs for *CXCR4*. (C) Docking model of C3 with CARBENOXOLONE. (D) Docking model of CXCR4 with CXCR4 ANTAGONIST USL311.

As a preliminary step toward exploring potential therapeutic hypotheses, we screened the Drug Gene Interaction Database (DGIdb) for candidate drugs targeting C3 and CXCR4. In this analysis, 9 putative compounds targeting *C3,* including CARBENOXOLONE, COMPSTATIN, and 24 compounds targeting *CXCR4,* such as CXCR4 ANTAGONIST USL311, PLERIXAFOR. In contrast, no targeted therapeutic agents were identified for *MEF2C* (Figs 9A and 9B).

To further assess the binding potential of these candidates, molecular docking was performed on the top-ranked compounds. CARBENOXOLONE-C3 and CXCR4 ANTAGONIST USL311-CXCR4 complexes yielded docking scores of −7.6 and −8.8 kcal/mol, respectively, exceeding the predefined significance threshold of −5.0 kcal/mol (Figs 9C and 9D; S15 Table). These in silico results suggest that carbenoxolone and the CXCR4 antagonist USL311 could be prioritized for future experimental testing in PD, but they do not establish therapeutic efficacy.

## Discussion

Emerging evidence positions immunometabolism as a critical nexus in chronic inflammatory diseases, yet its role in periodontitis remains underexplored. The pathogenesis of PD has traditionally been attributed to a dysregulated host response to a dysbiotic microbiome [5,7,46,47]. Our study expands the current view of periodontitis by identifying a fumarate-associated immunometabolic signature centered on C3, CXCR4, and MEF2C. These findings suggest a potential link between metabolic reprogramming and immune dysregulation in periodontal tissues, but do not establish a causal role for fumarate in disease pathogenesis. This interpretation is consistent with recent reports implicating fumarate accumulation in inflammatory programs in other chronic diseases [24,26,27], and extends these observations to the periodontal microenvironment.

The identification of C3 as a highly connected gene in the inferred network corroborates and extends prior genetic evidence. Importantly, Mendelian randomization (MR) and genetic inhibition experiments provide evidence for a causal role of *C3* in periodontitis, as genetic inhibition is associated with reduced disease risk [48,49]. Our findings place these prior observations in a cell-type-resolved context, showing that *C3* upregulation occurs in stromal fibroblasts and myeloid cells/neutrophils—cell populations known to participate in complement-mediated tissue injury in periodontitis. This cell-type specificity suggests that C3 may serve as a bridge between innate complement activation and adaptive immune responses, as reflected in its associations with plasma cell and naive CD4 + T cell infiltration [50,51]. Taken together, these observations suggest that fumarate-associated transcriptional alterations may coexist with *C3* upregulation in periodontitis; however, direct upstream regulation cannot be inferred from the present data.

The role of *CXCR4* in periodontitis has been implicated in previous studies examining chemokine-mediated leukocyte recruitment [52], but its association with metabolic dysregulation remains incompletely understood. *CXCR4*, which is highly expressed in T cells and plasma cells, regulates the trafficking and retention of diverse immune cells and promotes self-sustaining inflammation, a hallmark of chronic lesions [52]. Our single-cell analysis suggested that *CXCR4* expression is associated with predicted myeloid-T cell communication and relatively higher expression in later inferred myeloid/neutrophil trajectory states. This observation is biologically plausible because the CXCL12/CXCR4 axis is a key regulator of neutrophil bone marrow retention, release, and trafficking, and has also been implicated in T-cell migration and immune-cell recruitment [53–56]. This inferred trajectory pattern contrasts with *C3*, which showed relatively higher expression in earlier inferred states. *C3* is a central complement component involved in periodontal inflammation and complement-dependent activation of neutrophil/myeloid responses [48,57,58]. Together, these findings suggest that *CXCR4* and *C3* may be associated with distinct inferred myeloid/neutrophil states in PD, rather than forming a strict sequential causal hierarchy.

*MEF2C* emerged as another potentially informative component of the PD-associated transcriptomic feature set. While previous studies have linked *MEF2C* to NF-κB-mediated pathways in neuroinflammation [59], its role in periodontal disease remains poorly defined. Our data indicate that *MEF2C* is predominantly expressed in the stromal and vascular compartments, including VECs, LECs, and Pericytes/VSM, suggesting an association with stromal dysfunction and tissue remodeling. This interpretation is consistent with previous evidence showing that *MEF2C* is expressed in endothelial cells, smooth muscle cells, and surrounding mesenchyme during vascular development, and that MEF2C/MEF2 activity regulates endothelial function, vascular homeostasis, endothelial inflammation, and smooth muscle cell migration [60–65]. In periodontitis, single-cell and histological studies have further implicated endothelial cells, fibroblasts, and pericyte-like stromal populations in the inflammatory periodontal microenvironment, vascular remodeling, hypoxia, and impaired tissue homeostasis [66,67]. Given that experimentally established fumarate-related signaling is more commonly linked to Nrf2-mediated antioxidant responses than to *MEF2C* itself [68,69], *MEF2C* should be considered a candidate factor involved in stromal remodeling rather than a confirmed mediator of fumarate signaling.

Our single-cell transcriptomic analysis provides a systems-level view of how these three genes are organized within the remodeled periodontal microenvironment. The observed expansion of neutrophils, myeloid cells, and γδ T cells, coupled with contraction of stromal populations, mirrors the immunological shift reported in other chronic inflammatory conditions and supports the biological relevance of our findings. Notably, CellChat predicted a PD-enriched C3-(ITGAX+ITGB2) communication pattern between fibroblasts and myeloid/neutrophil cells. This computationally inferred intercellular communication pattern suggests that stromal-derived C3 may participate in myeloid cell activation, but dedicated functional experiments will be required to confirm this mechanism.

Our findings should also be interpreted within the broader inflammatory framework of periodontitis. Classical mediators such as IL-1β, TNF-α, and IL-6 are well-established contributors to periodontal tissue destruction [70,71]. Although these cytokines were not functionally examined in the present study, the C3/CXCR4/MEF2C-associated transcriptomic feature set identified here is better understood as operating alongside these canonical inflammatory pathways rather than replacing them. This interpretation is consistent with evidence that complement C3 contributes to periodontal inflammation and bone loss, CXCR4 participates in immune-cell recruitment and neutrophil dynamics in periodontitis, and MEF2C regulates endothelial inflammation and vascular homeostasis [48,64,72].

From a translational perspective, our in-silico screening identified C3- and CXCR4-targeting compounds that may serve as candidates for future experimental evaluation. However, the present data do not support immediate clinical application. The three genes should be considered candidate transcriptomic markers that may help prioritize mechanisms, cell populations, and intervention hypotheses for future work, but their clinical utility will require prospective validation in well-phenotyped cohorts, protein-level confirmation, and comparison with established periodontal diagnostic measures. Molecular docking alone does not establish biological efficacy, specificity, safety, or therapeutic benefit. Therefore, these results should be interpreted as hypothesis-generating observations rather than evidence supporting drug repurposing at this stage.

Several methodological considerations warrant discussion. First, the FRG panel was derived from a keyword-based GeneCards search and was intentionally used as a broad exploratory candidate set; as such, it may include genes with variable biological relevance to fumarate metabolism. Second, direct fumarate measurements and experimental manipulation of fumarate metabolism were not performed, so the present findings support association rather than causation. Third, biological validation was limited to a small pilot qRT-PCR experiment in a parallel-group ligature-induced mouse model, without protein-level, histologic, micro-CT, or functional validation. The animal experiment was conducted in male C57BL/6 mice using a 10-day ligature protocol selected for transcript-level validation; although longer 14- to 15-day protocols are frequently used to evaluate more established bone destruction, earlier inflammatory and bone-related changes can occur within the first week in mouse ligature models [40,73]. Differences in strain background, disease kinetics, complement activity, immune-cell composition, and endpoint selection between murine ligature models and human chronic periodontitis should therefore be considered when extrapolating these findings [73]. Fourth, although tribromoethanol was used consistently across the animal procedures, we acknowledge that injectable anesthetics may have systemic effects that could influence inflammatory readouts. Therefore, the qRT-PCR experiment should be interpreted as supportive transcript-level validation of the bioinformatic findings rather than as definitive functional evidence. Future studies using larger cohorts, alternative anesthetic regimens, histological and micro-CT validation, and protein-level assays will be required to confirm the biological relevance of these transcriptomic changes. Fifth, the public transcriptomic datasets lacked detailed clinical metadata, including probing depth, clinical attachment loss, bleeding on probing, smoking status, diabetes status, and disease staging/grading, which limits immediate clinical translation. Finally, the drug-screening results are purely in silico and require experimental validation of binding affinity, specificity, safety, and therapeutic efficacy. Future studies should incorporate metabolomic measurements, curated pathway-based FRG definitions, functional perturbation assays, prospective clinical cohorts, protein-level validation, standardized animal-model reporting, and experimental drug testing to clarify the biological and translational relevance of the proposed fumarate-associated transcriptomic feature set.

## Conclusion

In summary, this study identifies a fumarate-associated immunometabolic signature in periodontitis and highlights *C3*, *CXCR4*, and *MEF2C* as candidate transcriptomic markers linked to immune and stromal remodeling. By integrating bulk and single-cell transcriptomic analyses with machine-learning-based screening, our work provides a framework for future mechanistic studies. The animal validation and drug-screening results should be considered preliminary, hypothesis-generating findings that require prospective clinical validation, protein-level confirmation, and functional experiments before diagnostic or therapeutic implications can be drawn.

## Supporting information

S1 Fig Sample clustering and soft threshold selection for WGCNA. (A) Sample clustering dendrogram and trait heatmap. The left panel displays the hierarchical clustering tree of samples, and the bottom heatmap shows the sample-grouping trait (Control/PD, denoted by red/white blocks); (B) Soft threshold power selection plots. Left (Scale independence): The y-axis shows the scale-free topology fitting index, and the x-axis denotes the soft threshold power. Right (Mean connectivity): The y-axis represents the mean network connectivity; the curve shows that average connectivity decreases as the soft threshold increases.
(TIF)

S2 Fig Quality control and preprocessing of single-cell RNA-seq data. (A) Single-cell data before quality control (QC); (B) Single-cell data after QC; (C) Highly variable gene (HVG) selection; (D) Linear dimension chart of principal component analysis (PCA); (E) Scree plot of PCA; (F) PCA scatter plot of samples. Each point represents a single cell, and different colors denote distinct samples.
(TIF)

S3 Fig Single-cell clustering, cell type annotation, and group-specific expression analysis. (A) UMAP clustering plot of single cells. Different colors correspond to distinct cell types; (B) Dot plot showing the expression levels of signature genes for the eight annotated cell types; (C) Marker gene expression heatmap for cell-type annotation. Rows represent cell types, columns represent individual cells, and color intensity reflects the normalized expression of lineage-specific marker genes; (D) Box plots of cell types in Control (red) and PD (blue) groups. The x-axis denotes cell types, and the y-axis represents the relative proportion of each type; (E) Violin plots of key genes (CXCR4, C3, MEF2C) across cell types. The x-axis represents cell types, and the y-axis represents normalized gene expression) (F) Scatter plot of key gene expression in cell types; (G) Box plots of marker gene expression for major cell types in Control (blue) and PD (red) groups.
(TIF)

S4 Fig Cell-cell communication network remodeling between the control and periodontitis (PD) groups. (A) Cell-cell communication network of the Control group. The upper panel shows the communication-weight network; the lower panel shows the connection-count network; (B) Cell-cell communication network of the PD group. The upper panel shows the communication-weight network; the lower panel shows the connection-count network; (C) Heatmap of cell-cell interaction counts in the Control group. Rows/columns represent cell types, and color intensity denotes the number of ligand-receptor-mediated interactions between two cell types; (D) Heatmap of cell-cell interaction counts in the PD group. Rows/columns represent cell types, and color intensity denotes the number of ligand-receptor-mediated interactions between two cell types.
(TIF)

S5 Fig Subcluster analysis of Myeloid/Neut cells. (A) Scree plot of PCA; (B) PCA scatter plot of samples; (C) UMAP clustering plot of Myeloid/Neut subcluster.
(TIF)

S6 Fig Pseudotime trajectory and differentiation state analysis of Myeloid/Neut cells. (A) Diagram of pseudotime; (B) Trajectory maps of different cell subclusters; (C) Different stages of differentiation trajectory; (D) Different stages of differentiation trajectory in Control and PD groups; (E) Trajectory maps of key gene expression; (F) Dynamic heat map of key gene expression. It shows the gene expression profile differences among cells at different differentiation states along the pseudotime trajectory, with the color gradient representing expression levels.
(TIF)

S1 Table Differentially expressed genes between control and periodontitis groups in bulk RNA-seq data. (XLSX)

S2 Table GO and KEGG enrichment analysis of differentially expressed genes in bulk RNA-seq data. (XLSX)

S3 Table Core module genes of periodontitis in WGCNA. (XLSX)

S4 Table Fumarate-related genes in the GeneCards database. (XLSX)

S5 Table Key genes related to fumarate and PD by Venn diagram analysis. (XLSX)

S6 Table GO and KEGG enrichment analysis of key genes related to fumarate and PD. (XLSX)

S7 Table Key transcriptomic markers associated with fumarate and PD were identified by machine learning. (XLSX)

S8 Table Primer used for analyzing key genes. (XLSX)\

S9 Table Immune infiltration analysis in control and PD. (XLSX)

S10 Table Correlation analysis of key genes. (XLSX)

S11 Table GSEA of key genes. (XLSX)

S12 Table CeRNA network analysis of key genes. (XLSX)

S13 Table Distribution of cell numbers per cell type in control and PD groups of scRNA. (XLSX)

S14 Table Cell-cell communication of cell types in control and PD groups of scRNA. (XLSX)

S15 Table Screening of drugs with key genes in PD. (XLSX)

S1 File Humane endpoints checklist. (DOCX)

S2 File Source data. (XLSX)

## Abbreviations

TCA: Tricarboxylic acid
AUC: Area under the curve
PD: Periodontitis
HIF-1α: Hypoxia-inducible factor 1-alpha
FRGs: Fumarate-related genes
DEGs: Differentially expressed genes
FC: Fold change
WGCNA: Weighted gene co-expression network analysis
TOM: Topological overlap matrix
MM: Module membership
GS: Gene significance
LASSO: Least absolute shrinkage and selection operator
SVM-RFE: Support vector machine recursive feature elimination
UMAP: Uniform manifold approximation and projection
GO: Gene Ontology
KEGG: Kyoto Encyclopedia of Genes and Genomes
CAMs: Cell adhesion molecules
PPI: Protein-Protein Interaction
DCs: Dendritic cells
Tfh: follicular helper T
Treg: regulatory T
GSEA: Gene Set Enrichment Analysis
ceRNA: competing endogenous RNA
Myeloid/Neut: Myeloid/Neutrophils
Pericytes/VSM: Pericytes/Vascular smooth muscle cells
LECs: Lymphatic endothelial cells
VECs: Vascular endothelial cells
MR: Mendelian randomization
